# Culture-Independent Detection and Identification of *Leptospira* Serovars

**DOI:** 10.1128/spectrum.02475-22

**Published:** 2022-11-29

**Authors:** Michael A. Matthias, Aristea A. Lubar, Shalka S. Lanka Acharige, Kira L. Chaiboonma, Nicholas N. Pilau, Alan S. Marroquin, Dinesha Jayasundara, Suneth Agampodi, Joseph M. Vinetz

**Affiliations:** a Department of Medicine, Division of Infectious Diseases, University of California, San Diego, California, USA; b Leptospirosis Research Laboratory, Department of Community Medicine, Faculty of Medicine and Allied Sciences, Rajarata University of Sri Lanka, Mihintale, Sri Lanka; c Department of Internal Medicine, Section of Infectious Diseases, Yale School of Medicine, New Haven, Connecticut, USA; d Faculty of Veterinary Medicine, Usmanu Danfodiyo University, Sokoto, Nigeria; e Department of Microbiology, Faculty of Medicine and Allied Sciences, Rajarata University of Sri Lanka, Mihintale, Sri Lanka; f Department of Community Medicine, Faculty of Medicine and Allied Sciences, Rajarata University of Sri Lanka, Mihintale, Sri Lanka; University of Wisconsin-Madison

**Keywords:** classification, diagnostics, *Leptospira*, leptospirosis, serotyping, serum samples, urine samples, qPCR

## Abstract

Pathogenic *Leptospira*, the causative agents of leptospirosis, comprise >200 serotypes (called serovars). Most have a restricted reservoir-host range, and some, e.g., serovar Copenhageni, are cosmopolitan and of public health importance owing to their propensity to produce severe, fatal disease in humans. Available serotyping approaches—such as multilocus sequence typing, core genome sequence typing, pulsed-field gel electrophoresis, and the cross-agglutination absorption test—are tedious and expensive, and require isolation of the organisms in culture media—a protracted and incredibly inefficient process—precluding their use in prospective studies or outbreak investigations. The unavailability of culture-independent assays capable of distinguishing *Leptospira* serotypes remains a crucial gap in the field. Here, we have developed a simple yet specific real-time qPCR assay—targeting a *Leptospira*-unique gene encoding a putative polysaccharide flippase—that provides intraspecies, serotype-defining (i.e., epidemiologically useful) information, and improves upon the sensitivity of preferred *lipL32*-based qPCR-based diagnostic tests. The assay, dubbed RAgI (“rage one”), is rapid and affordable, and reliably and specifically detects group I pathogenic *Leptospira* in culture, serum, and urine, with no detectable off-target amplification—even of the genetically related but low virulence group II pathogenic (formerly “intermediate”) or nonpathogenic *Leptospira*. It retained 100% diagnostic specificity when tested against difficult sample types, including field-collected dog urine samples and environmental samples containing varied and complex microbial species-consortia. This assay holds considerable promise in the clinical setting, and for routine epidemiological and environmental surveillance studies.

**IMPORTANCE** Leptospirosis is caused by a diverse group of pathogenic spirochetes comprising over 200 different serotypes. Some are widely reported and of public health importance owing to their propensity to produce severe, fatal disease in humans. Apart from their tedium and expense, current serotyping approaches require isolation of the organisms in culture media—a protracted and incredibly inefficient process—rendering them useless clinically and limiting their utilization in prospective studies or outbreak investigations. The unavailability of culture-independent assays capable of distinguishing *Leptospira* serotypes remains a crucial gap in the field. The 11108 qPCR-assay overcomes this barrier to progress via direct taxonomic and serotype classification of *Leptospira* from urine and serum samples, and hence, is the first qPCR-based prognostic test for human leptospirosis.

## INTRODUCTION

Leptospirosis is a sometimes lethal, waterborne infectious disease caused by pathogenic *Leptospira* (1–4). Humans are usually infected via contact with contaminated surface water or damp soil, but occupational exposure in kennels, slaughterhouses, and animal farms also occurs, albeit relatively infrequently. Rodents are notorious “reservoir” hosts, but virtually any terrestrial species, including pets and domesticated animals, can carry and disseminate *Leptospira*. Transmission is presumably more common in warm, wet locales, but disease is cosmopolitan with a global burden comparable with cholera and typhoid fever (5, 6). Epidemics grow more frequent, made worse by changing weather patterns and increased overcrowding in urban slum communities (5–7). Even so, the scarcity of epidemiological data (well-curated or otherwise), insufficient public health awareness, extensive evidence gaps, and a lack of adequate diagnostic tests that, collectively, artificially depress incidence rates, indicate that the public health impact of leptospirosis is greatly underappreciated. Yet, despite several well-documented limitations, mainly their inability to provide definitive results soon enough for clinical interventions to be effective (8), antibody-based diagnostic tests (ABTs), e.g., the Microscopic Agglutination Test (MAT) or IgM-based ELISAs remain the norm, only worsening the public health outlook.

Newly developed leptospirosis diagnostic assays have tended to focus instead on antigen and nucleic acid detection. Of the latter, most are nucleic acid amplification-based diagnostic tests (NAATs) that target various leptospiral genes and are designed to provide an early diagnosis of leptospirosis (9–14). In practice, many have context-related limitations, particularly a low diagnostic sensitivity when epidemiological information is lacking; therefore, few are generalizable. Leptospirosis NAATs-du-jour target *lipL32* (15–17), a taxonomically restricted gene found almost exclusively in *Leptospira*. *Leptospira* alleles are distinctive and are found only in pathogenic species, ergo *lipL32*-based assays are specific for medically important *Leptospira*. They tend to be remarkably consistent (dynamic range of 10^7^ to 10^0^ [15]) but produce somewhat unpredictable results with non-L. interrogans leptospiral species, owing to the high sequence diversity of the targeted gene. Despite these recognized benefits, *lipL32*-based assays are not yet routine in clinical or public health settings. Presumably because the best options employ fluorescent probes, which inflate setup and operational costs, rendering them ill-suited to the low-tech realities of many leptospirosis endemic regions. To expand access, less expensive PCR-based assays have been developed (18), however, these by their nature suffer from inferior (i.e., higher) detection limits and are tedious compared with qPCR-based alternatives. Regrettably, *lipl32*-based NAATs provide little insight with respect to infecting *Leptospira* species or serotype. Accordingly, they are best suited to the clinical setting where an early diagnosis is paramount.

Some leptospirosis NAATs allow species identification (ID), e.g., *OmpL1*-PCR (19), but a universal limitation of these assays has been their inability to provide serotype information. Serovar ID remains both clinically and epidemiologically useful, as some serovars, e.g., Copenhageni (shorthand notation, *sv*Copenhageni), are especially virulent, and many have preferred reservoirs, e.g., *sv*Copenhageni for rats, and *sv*Canicola and *sv*Hardjo for dogs and cattle, respectively (1). Reputed “gold standard” approaches for serotyping *Leptospira* strains, such as the Cross Adsorption Agglutination Test (CAAT), and its modern equivalent utilizing panels of cross-reacting monoclonal antibodies (MAbs), are complicated, expensive, and impractical. Nucleic Acid Based Serotyping (NABS) methods, such as multi-locus sequence typing (MLST) (20, 21), multi-locus variable number tandem repeat analysis (MLVA) (22), and, more recently, core genome multi-locus sequence typing (cgMLST) (23) have limited clinical utility due to their reliance on culture isolation (as do all current *Leptospira* serotyping methods) and are impractical in most epidemiological contexts. Indeed, several NABS-rooted schemes have been proposed and abandoned for varied reasons but primarily their inconsistent performance with non-L. interrogans species. And while a newer qPCR based MLVA approach employing high resolution melt analysis (HRMA) is promising (24), its complexity and cost has limited its application (25). Consequently, development of inexpensive, easily deployable, culture-independent, and antigen-detection tests capable of distinguishing *Leptospira* serovars is paramount.

Here, we report on the development and validation of the first rapid affordable group I-specific (RA*g*I) NAAT capable of reliably distinguishing *Leptospira* serotypes in diverse sample types, obviating requirements for culture isolation. The assay, which we dubbed the leptospirosis 11108 RA*g*I (or “rage-one”) test, targets LIC_RS05715 (old locus_tag: LIC_11108 [abbreviated 11108 hereafter])—a distinctive, single-copy, leptospiral core gene encoding a putative oligosaccharide flippase (NCBI protein ID: AAS69715.1; UniProtKB ID: Q72TB3)—and allows joint inference of *Leptospira* species and serovar. The data presented here provide crucial proof-of-concept validation for and should spur development of conceptually related leptospirosis serotyping NAATs, (i.e., sNAATs).

## RESULTS

### Detection limit and cross-species amplification of deconvoluted (i.e., species-defined) 11108 primer sets.

For most species, the optimized 11108-assay had a detection limit of 10 GEs with expected T_m_ regardless of diluent (i.e., PBS or NHS). However, to achieve a similar level of detection with *borgpetersenii*, and *santarosai*-specific primer pairs, extended runs (>40 to 45 cycles) were required. Under optimized assay conditions, the detection of the *borgpetersenii*, and *santarosai*-specific primers was 10^2^ to 10^3^ GEs.

### Serovar resolution via traditional block-based thermal cycling.

As summarized in Table 1, CFX96-generated T_m_’s ranged from 80°C to 85°C. Negative controls and NTCs were invariably negative, confirming specific detection of group I pathogenic *Leptospira*. Intra- and inter-run T_m_’s were remarkably consistent (*σ* = 0.0°C) among replicates, with T_m_’s defined for each allele (Table 1). At the manufacturer-recommended resolution of 0.2°C, nine *interrogans*/*kirschneri* sequence variants or alleles produced only four T_m_’s, ranging from 80.2 to 80.8°C, though all could be differentiated at 0.1°C-resolution (not shown). Nonetheless, several important serovars could be distinguished based on 11108-amplicon derived T_m_’s, including *sv*Copenhageni/*sv*Icterohaemorrhagiae (preferred hosts = peri-domiciliary rats), *sv*Lai (field mice), *sv*Hardjo-prajitno (cattle), and *sv*Canicola/*sv*Pomona (dogs and pigs, respectively). T_m_’s were species-dependent, as expected based upon species-variable GC%. With *interrogans*, *kirschneri*, and *noguchii* alleles producing T_m_’s ranging from 8°C to 81°C; *borgpetersenii*, 81.°C to 82.5°C; *weilii*, 83°C; and *santarosai*, >84°C (Table 1; Fig. S2); joint inference of species and serotype could be made. Therefore, *interrogans sv*Hardjo subtype *hardjoprajitno* and *borgpetersenii sv*Hardjo *bovis* could be readily distinguished.

**TABLE 1:** T_m_’s produced by various *Leptospira* strains, including isolates from Peru and Sri Lanka

Species	Serogroup^*a*^	Serovar^*b*^	GC%	CFX96-T_m_/^o^C^*c*^	SD	▿MIC-T_m_/^o^C	SD	Allele^*g*^	
*interrogans*	Australis	*sv*Australis	39.5	**80.8**	±0	**81.87**	±0.004	**AG1**	**—**
		*sv*Bratislava	39.5	80.8	±0	**—**	**—**	**AG1**	**—**
	Autumnalis	*sv*Autumnalis	39.5	**80.8**	±0	**81.88**	±0.004	**AG2**	
		*sv*Weersinghe^*c*^	38.7	80.6	±0	**ND** ^*h*^	**—**	**AG3**	**SL-1**
	Bataviae	*sv*Bataviae^*d*^	38.3	**80.2**	±0	**81.19**	±0.005	**AG4**	**PER-1**
	Canicola	*sv*Canicola	39.5	**80.8**	±0	**81.83**	±0.010	**AG5**	**PER-2**
	Djasiman	*sv*Djasiman	39.5	80.8	±0	**—**	**—**	**AG1**	**—**
	Grippotyphosa	*sv*Grippotyphosa	39.5	80.8	±0	**—**	**—**	**AG1**	**—**
	Icterohaemorrhagiae	*sv*Copenhageni^*d*^	38.7	80.2	±0	**81.22**	±0.000	**AG6**	**PER-3**
		*sv*Icterohaemorrhagiae^*d*^	38.7	80.2		**—**	**—**	**AG6**	**PER-3**
		*sv*Lai	38.3	80.4	±0	**81.56**	±0.005	**AG7**	**—**
		*sv*Mankarso	39.1	**80.8**	±0	**81.76**	±0.005	**AG8**	**—**
	Pomona	*sv*Pomona	39.5	80.8	±0	**ND**	**—**	**AG5**	**—**
	Pyrogenes	*sv*Manilae	39.5	**80.8**	±0	**81.79**	±0.022	**AG9**	**—**
		*sv*Pyrogenes^*f*^	39.5	**80.8**	±0	**ND**	**—**	**AG10**	**SL-2**
	Sejroe^a^	*sv*Hardjo	38.3	**80.6**	±0	**81.72**	±0.000	**AG11**	**—**
		*sv*Wolffi	38.3	**80.6**	±0	**—**	**—**	**AG11**	**—**
		*sv*Geyaweera^*c*^	—	**ND**	**—**	**ND**	**—**	**—**	**—**
*kirschneri*	Cynopteri	*sv*Cynopteri^*d*^	39.5	**80.2**	±0	**81.28**	±0.004	**AG12**	**PER-4**
	Grippotyphosa	*sv*Ratnapura^*c*^	39.9	80.4	±0	**ND**	**—**	**AG13**	**SL-3**
*noguchii*	Australis	*sv*Peruviana^*d*^	—	**IA**	±0	**ND**	**—**	**—**	**—**
*borgpetersenii*	Ballum	*sv*Ballum	42.9	81.6	±0	**ND**	**—**	**GTG1**	**—**
	Javanica	*sv*Javanica	42.9	81.6	±0	**ND**	**—**	**GTG2**	
		*sv*Ceylonica^*c*^	42.9	81.4	±0	**ND**	**—**	**GTG3**	**SL-4**
	Sejroe	*sv*Hardjo	44	82.2	±0	**ND**	**—**	**GTG4**	**—**
	Tarassovi	*sv*Tarassovi	44	82.0	±0	**ND**	**—**	**GTG5**	**—**
*weilii*	Celledoni	*sv*Celledoni	45.5	82.8	±0	**ND**	**—**	**GCG1**	**—**
*santarosai*	Autumnalis	*sv*Alice^*c*^	48.9	85.0	±0	**ND**	**—**	**GCA1**	**SL-5**
	Bataviae	*sv*Rioja^*d*^	—	**IA**	**—**	**ND**	**—**	**—**	**—**
	Cynopteri	*sv*Tingomaria^*d*^	—	**IA**	**—**	**ND**	**—**	**—**	**—**
	Hebdomadis	*sv*Borincana	48.9	84.8	±0			**GCA2**	**—**
	Javanica	*sv*Vargonicas^*d*^	—	**ND**	±0	**ND**	**—**	**—**	**—**
	Mini	*sv*Georgia	48.9	84.8	±0	**ND**	**—**	**GCA2**	
		*sv*Ruparupae^*d*^	—		±0	**ND**	**—**	**—**	**—**
	Pyrogenes	*sv*Alexi	49.2	85.0	±0			**GCA3**	**—**
		*sv*Bagua^*d*^	—	**IA**	**—**	**ND**	**—**	**—**	**—**
		*sv*Cenepa^*d*^	—	**IA**	**—**	**ND**	**—**	**—**	**—**
	Sarmin	*sv*Machiguenga^*d*^	—	84.9	**—**	**ND**	**—**	**GCA4**	**—**
	Shermani	*sv*Shermani	48.9	85.0	±0	**—**	**—**	**GCA5**	**—**
*licerasiae*	Varillal	*sv*Varillal^*e*^	—	**NA**	**—**	**NA**	**—**	**—**	**—**
*wolffii*	Undetermined	*sv*Khorat^*e*^	—	**NA**	**—**	**NA**	**—**	**—**	**—**

a Serogroup in bold; ^a^ classification uncertain for *sv*Geyaweera.

b Serovars named via shorthand, e.g., *sv*Icterohaemorrhagiae **=** serovar Icterohaemorrhagiae—for clarity and to avoid confusion with Serogroup designations.

c Serovars from Sri Lanka (36–38).

d Serovars reported from Peru (39, 40).

e Group II serovars used as specificity controls.

f T_m_’s produced using manufacturer-recommended 0.2°C increments for melt-curve analysis—values in bold resolved using 0.1°C increments or on MIC. **(▿)** T_m_ **≅** CFX-T_m_ + 1.2°C.

g Targeted regions (“11108-alleles”) in some serotypes are identical, e.g., *interrogans sv*Copenhageni/*sv*Icterohaemorrhagiae, *sv*Canicola/*sv*Pomona, and *sv*Hardjo/*sv*Wolffi; alleles found in Peru and Sri Lanka are indicated and have been given a numeric identifier (e.g., PER-1 and SL-1, respectively).

h ND, not done; IA, inconsistent amplification, indicating suboptimal (primer) annealing and concomitantly, reduced qPCR amplification efficiency; NA, no amplification.

We also analyzed reference strains obtained from Peru and Sri Lanka, where we have conducted prospective field studies. All strains originating from Sri Lanka (*sv*Geyaweera**/***sv*Weersinghe, *sv*Ratnapura, *sv*Ceylonica, and *sv*Alice) could be reliably differentiated from each other (80.6°C, 80.4°C, 81.6°C, 85°C), and from *sv*Copenhageni**/***sv*Icterohaemorrhagiae (80.2°C). In contrast, Peruvian reference strains originating from outside Iquitos, specifically those belonging to *L. santarosai*, produced inconsistent melt peaks, precluding confident T_m_ assignment, which is likely due to reduced primer annealing and, thus, amplification efficiency, from these (as-yet) unknown sequence variants, suggesting that alternative forward and/or reverse primers might be needed (as was the case for some *interrogans* [e.g., *svManilae*] and *noguchii* strains).

### Serovar resolution using high-precision magnetic induction thermal cycling.

Owing to a different design, MIC-derived T_m_’s were on average ~1°C higher than those produced by the CFX96 but were also reliable (*σ* ≤ 0.05). Statistical comparison of the means showed that the superior precision of the MIC improved allele resolution (up to 100 potential distinct T_m_’s at 0.05°C resolution, and 200 at 0.025°C resolution). Accordingly, previously unresolved (but distinct) alleles in *sv*Bataviae, *sv*Copenhageni**/***sv*Icterohaemorrhagiae, *sv*Cynopteri, *sv*Hardjo-prajitno**/***sv*Wolffi, *sv*Mankarso, *sv*Australis**/***sv*Bratislava*/sv*Djasiman/*sv*Grippotyphosa/*sv*Pyrogenes, *sv*Autumnalis, *sv*Canicola**/***sv*Pomona, and *sv*Manilae were assigned distinctive T_m_’s (Table 1). Overall, all nine *interrogans*/*kirschneri* sequence variants could be reliably distinguished based upon mean T_m_’s (*P* < 0.05).

### Reliability, accuracy, sensitivity, and specificity of 11108-based assay.

During the 5-year-period of December 2002 to June 2007, 144 *Leptospira* strains were isolated from humans (50), peri-domiciliary rats (34), livestock (32), and wild animals (28) in Iquitos and surrounding areas. The majority (65.3%) were either *interrogans*, *kirschneri*, or *noguchii*. Fifty (34.7%) were identified as *santarosai*, with 27 (or 54%) derived from livestock (i.e., buffaloes, cattle, and pigs) representing 84.4% of all isolates obtained from these sources. In contrast, only 14 human isolates were classified as *santarosai* (28%), as were six from wild animals (21.4%), two from rats (5.9%), and none from bats or dogs. To test the reliability and accuracy of our optimized 11108-based NAAT, we analyzed a sample of these isolates, inclusive of all species and Serogroups found in the region. Twenty-three were taxonomically classified as *interrogans*, 13 as *santarosai*, seven as *L*. *noguchii*, and two as *L*. *kirschneri*. Table 2 summarizes the 11108 qPCR-assay results of all human and rat isolates analyzed, each producing a reliable T_m_.

**TABLE 2:** *Leptospira* strains isolated over a 5-year period (2002 to 2007) from humans and peri-domiciliary rats in Iquitos, Peru^*a*^

Source (# Isolates)	Species	CAAT/pFGE	T_m_/^o^C	Allele^*b*^	Consistency (%)	Dates MM/YY	Age/yrs	Agreement
Human (28)	*interrogans*	*sv*Bataviae	80.2	AG4	1/1	01/07	11	1/1
		*sv*Canicola	80.6	AG5	4/4	03/03 to 07/04	14 to 15	4/6
		*sv*Copenhageni	80.2	AG6	9/9	03/03 to 03/07	11 to 15	9/9
	*noguchii*	Australis	80.4	AA1	1/1	04/04	14	1/1
		Bataviae	80.4, 80.6, 80.8, 81.0	AA1, AA2, AA3, AA4	1/1, 3/3, 2/2	07/03 to 06/07	11 to 15	6/6
	*santarosai*	Pyrogenes	85.0	GCA6	1/1	12/04	14	1/1
		Tarassovi	84.8	GCA7	4/4	05/04 to 04/07	11 to 14	4/4
Rat (6)	*interrogans*	*sv*Copenhageni	80.2	AG4	3/3	12/02 to 02/03	15 to 16	3/6

a CFX96-derived T_m_ and relevant serotyping info are indicated (minimum of three replicates).

b AG alleles amplify with 11108*f*_**AGA** forward primer but not *_**AAA** forward, whereas AA alleles amplify with *_**AAA** forward but not *_**AGA**.

Based on CAAT (and/or PFGE-fingerprinting), 23 *interrogans* strains belonged to either serogroup Icterohaemorrhagiae (with all but two definitively typed as *sv*Copenhageni) or Canicola (*sv*Canicola, and two closely related but seemingly distinct strains, BEL039 and CBC1203R). All produced consistent T_m_’s which were verified by BLAST. Thus far, only two strains isolated in Iquitos, HAI1536 and MOR069, have been shown to have similar 11108 amplicons but different CAAT or PFGE fingerprints among the 19 analyzed that were classified as *interrogans/kirschneri/noguchii*. Similarly, isolate MMD4803—presumed to be distinct based on PFGE fingerprinting—could not be resolved from either of two HAI1378-derived isolates, nor CBC621. Overall, of the 33 isolates analyzed, only two produced 11108-inferred IDs that disagreed with CAAT- or PFGE-based assignments, indicating correct inferences approximately 94% of the time, regardless of isolation date or source (human, clinical versus mammalian reservoir). Species inferences were reliable, and the assay correctly differentiated alleles/sequence variants 100% of the time. As is the case for most “exotic” serovars, sequences were not publicly available for Bataviae strains, VAR132 (80.4°C), MOR069 (80.6°C), nor Shermani strains, HAI1378(U) and CBC621 (84.8°C), precluding definitive serovar assignment. However, these were all confirmed via BLAST best-hits to be distinct and derived from 11108-amplicon variants, and based upon T_m_-defined species-ranges, grouped with other serotypes from the expected species, i.e., *noguchii* and *santarosai*, respectively. Taken together these findings indicate that the 11108-assay is highly reliable and capable of producing consistent results with strains isolated several years apart and originating from various hosts. Therefore, although T_m_ values might vary based upon hardware/software options utilized, our approach consistently distinguished sequence variants.

**FIG 1:**
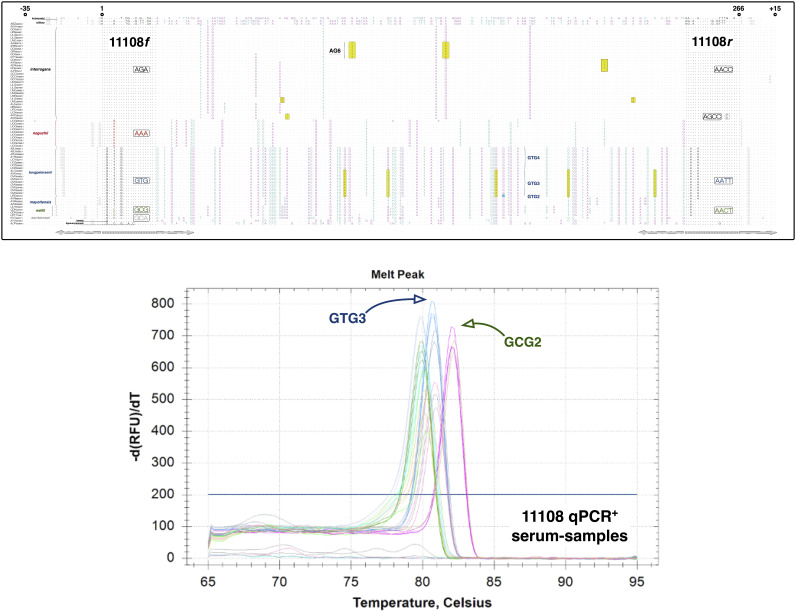
Schematic showing predicted and experimentally validated 11108-amplicon sequence-variants in patient sera. (Top panel) Global alignment of 266-bp region amplified by 11108 primer pairs with additional nucleotides at the 5′ and 3′ end, (+35 and −15, respectively). Forward (i.e., 11108f) and reverse primers and their species-associated sequence variants, e.g., 11108f_AGA for *interrogans*/*kirschneri* and *noguchii* strains, have been indicated. *Arrows* below MSA indicate alternate primer sites. Examples of allele defining SNPs are enclosed in yellow boxes. AG6 = *sv*Copenhageni. GTG3 = *borgpetersenii*, unknown (UNK) serogroup (i.e., no agglutination in serotyping reactions). GCG2 = *weilii* UNK serogroup. AG6 is dominant human infecting strain in Iquitos (Peru), whereas GTG3 and GCG2 comprise novel *noninterrogans* serovars isolated from hospitalized patients in Sri Lanka—Table S3. *Leptospira* spp. shown: *kobayashi* (top, nonpathogen), *biflexa* (nonpathogen), *interrogans*, *noguchii*, *borgpetersenii*, *mayottensis*, *weilii*, *santarosai*, *kmetyi* and *tipperaryanensis* (bottom). (Bottom panel) CFX96-generated melt-curves (without Precision Melt Software), showing T_m_’s and allele assignments from six (of 12) qPCR-positive serum-samples. Three species were reliably distinguished: *interrogans* (unlabeled peak), *borgpetersenii* GTG3, and *weilii* GCG2, consistent with predicted allele assignments and 11108-based amplification of cognate isolates.

Pilot experiments using archived culture-positive patient serum-samples from Sri Lanka (12 isolates, sample age range = 3 to 5 years old) and Iquitos (25, sample age range 11 to 15 years old) produced mostly consistent results. Ten of 12 recently archived serum-samples were 11108 qPCR positive (~83% sensitivity). Eight produced consistent amplification (i.e., were positive ≥67% of the time) across replicates and repeat runs (minimum of nine replicates), and two amplified less consistently, being positive <67% of the time. That said, all produced expected T_m_’s with respect to their cognate isolate. Fig. 1 shows melt-curves from six of these archived samples. Amplification of older samples (i.e., 11 to 15 years old) was less reliable, although all were positive with amplification evident in at least one replicate in all runs (i.e., ~33%). And regrettably, most produced indefinite or quasi-stable T_m_’s (±0.1°C), though some, e.g., serum-sample PAD451 (81.0°C), produced reliable T_m_’s agreeing with CAAT-based classification of its cognate isolate, indicating that further refinement of our assay could allow retrospective analysis of bio banked serum-samples.

Forty-four dog urine samples were initially classified as culture negative, leading to the presumptive identification of 11 false-positive samples (culture negatives that were positive by at least one real-time qPCR assay). Because culture is inefficient (<10% success rate, in our hands), these presumed false positives were verified to contain *Leptospira* DNA by shotgun metagenomics sequencing (SMS). All were positive by 11108-based real-time qPCR, of which four were also positive by *lipL32* qPCR (not shown). Partial *Leptospira* genomes were reconstructed from all but two, which produced mostly low-quality reads. Of the initial 84 samples, complete data were produced for 80: 47 verified positives and 33 negatives. Assay results were used to construct a contingency table to assess sensitivity and specificity (Table 3). The sensitivity (true positives detected) was clearly superior to the *lipL32*-based assay used for comparison while maintaining 100% specificity.

**TABLE 3:** Comparison of the sensitivity and specificity of 11108 and *lipL32* qPCR assays

Assay	Sensitivity	Specificity
11108	91.4%	100%
*lipL32*	68.1%	100%

## DISCUSSION

Recognizing the public health importance of real-time serovar ID, we developed a novel single-tube NAAT capable of providing reliable *Leptospira* serovar predictions directly from diverse sample types. Rigorous validation against dog urine samples proved our assay to be highly specific for group I pathogenic *Leptospira*, with no detectable off-target amplification of even genetically related (but usually hypovirulent) group II species or of unrelated, poorly characterized microorganisms in complex microbial species-consortia. It dramatically outperformed a mainstream *lipL32*-based alternative and proved unique in its capacity to provide joint species and serotype IDs, while consistently detecting fewer than 10 genome equivalents/sample in serum and urine. From a public health perspective, the early successes of our approach provide pivotal proof-of-concept validation that should spur development of a newer class of NAATs specifically for the detection and direct identification of *Leptospira* serovars in varied clinical settings and epidemiological contexts.

The MAT was formerly the gold standard for leptospirosis diagnosis. But, owing to well-documented limitations (and antibody-based diagnosis in general), it has been steadily supplanted by a bevy of NAATs, with *lipL32*-based real-time qPCR assays now routine. However, until now, NAATs by design could not generate epidemiologically useful information. Some 16S rRNA-based assays yield species IDs (18), but these alone provide limited epidemiological information, as many important but distinct serovars belong to the same species, making it nigh impossible to assess the relative importance of potential reservoir species, domesticated or otherwise, in the maintenance and transmission of human leptospirosis. Furthermore, as diagnostic tests, 16S rRNA-based assays, although sensitive, are mostly genus-inclusive (and not specific for pathogenic *Leptospira*); they tend to produce false-positive results, and are practically unusable with certain sample types, such as urine (26). In contrast, our assay and *lipL32*-based alternatives are highly specific for pathogenic species, but unlike the latter, ours proved to be as sensitive as those targeting the 16S rRNA gene. Moreover, of the three design philosophies, only our assay yielded joint predictions of infecting species and serovar. Therefore, based on its validated sensitivity and specificity, empirically determined detection limit, and the verified accuracy of its predictions, this new NAAT holds considerable promise for real-time serovar ID, an important milestone for public health leptospirosis surveillance programs.

We also introduce the concept of “sequence-variant allied” or “allele-specific” Tm’s (i.e., melt-temperatures), which we use interchangeably and show that under optimal conditions all unique 11108-amplicon variants could be reliably differentiated by this metric alone. More importantly, assay predictions were reliable (i.e., unexpected Tm’s were all well-supported by SNPs in sequenced amplicons), improving upon the MAT, which is currently the only other diagnostic test permitting inference of infecting serovar.

More importantly, assay predictions were reliable (i.e., unexpected T_m_’s were all well-supported by SNPs in sequenced amplicons), improving upon the MAT, which is currently the only other diagnostic test permitting inference of infecting serovar. Sadly though, MAT-based predictions are mostly inaccurate (27, 28) or ambiguous (29), yet presumably improved through analysis of paired (acute and convalescent) sera. As far as we know, the two most medically important serovars worldwide, L. interrogans *sv*Copenhageni and *sv*Canicola, were easily and reproducibly distinguished, as were others with shared host preferences. Even multispecies serovars, such as *sv*Hardjo *interrogans* subtype prajitno and *borgpetersenii* subtype bovis, were easily distinguished, as were novel or poorly characterized serovars with little to no genome information, which could be differentiated from each other (and from common serovars) based on T_m_, or amplicon-derived sequence data (as was the case with buffalo isolate, CBC621). As more genomes are sequenced and additional sequence variants are identified, further improvements or customizations are not only possible but easily developed. Even so, LIC_11108 is but one of many leptospiral genes we have identified (so far) that have clinical utility as targets of rapid diagnostic and/or prognostic tests.

Our approach is not intended to replace specialized serotyping methods, such as MLST profiling or PFGE fingerprinting, which by design have superior resolving power, but rather to address a clear need for NAATs capable of rapid and reliable serovar ID directly from varied sample types. NAATs that are readily adapted to diverse clinical settings and public health surveillance initiatives. Additionally, in recent years, emphasis has been placed upon bridging the gap between historical knowledge of *Leptospira* serological and molecular techniques, with growing concern that crucial epidemiological information will be lost in the transition to modern rapid diagnostics (30). We believe that our assay (and others adopting similar design principles) could ultimately bridge this gap.

## MATERIALS AND METHODS

### Primer design and validation.

For real-time qPCR-based serovar identification (ID), genes comprising the core *Leptospira* genome were first defined using the Pan-Genome Ortholog Clustering Tool, PanOCT (31), and those predicted to encode proteins participating in LPS assembly or export, e.g., O-antigen polymerases (*rfbY*), O-antigen/polysaccharide flippases, *rfbX* and LIC_11108, and the *Leptospira* lipopolysaccharide export system (Lpt), including *msbA*, *lptA*, and *lptD*, were used to construct exhaustive amino-acid-guided multiple sequence alignments (MSAs) of coding sequences (CDS) retrieved from GenBank—anticipating that these would be serogroup- and (potentially) serovar-dependent, due to their stereospecificity (32). LIC_11108 was chosen for initial evaluation based on several criteria (the most notable summarized in the preceding paragraph). From these MSAs, hypervariable regions were identified and targeted for amplification by primers complementary to conserved flanking sequence (Fig. S1).

As none of these flanking regions were 100% conserved, degenerate primers 11108*f*: 5′–TTR AAN GAR AGT ATA AAA CTT CC–3′, and 11108*r*: 5′–AAC GGR CTY TTT CAA TAY GC–3′ (Table 4)—specific for group I pathogenic *Leptospira*—were synthesized. Pilot experiments were conducted using 11108*f* and 11108*r* to confirm amplification of globally distributed *Leptospira* species: L. interrogans, *L. kirschneri*, *L. noguchii*, L. borgpetersenii, *L. weilii*, and *L. santarosai*. Amplification of *L. mayottensis*, which has a more restricted distribution, was assessed via *in silico* PCR of available genome sequences. Baseline assay conditions were established using degenerate primers, following which optimal conditions were determined using deconvoluted species-specific primers (Table 4). Cognizant of our intended longer-term development goals, we opted for an affordable SYB green-based NAAT that could be easily customized for probe-based assays (should the need arise) and evaluated its performance on affordable hardware/software combinations, including a four-channel magnetic induction thermal cycler (MIC) manufactured by Bio Molecular Systems (~40% cost of CFX96).

**TABLE 4:**
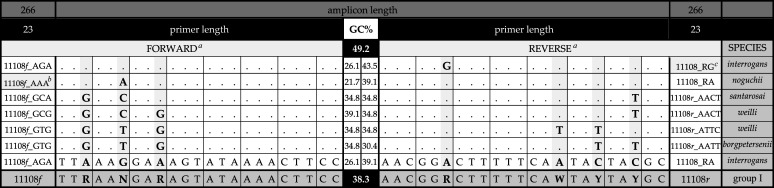
11108 qPCR assay, forward and reverse primer pairs

^a^The 11108*f*_***AGA***/11108*r*_***RA*** primer pair should amplify most L. interrogans, *L*. *kirschneri*, and *L*. *noguchii* alleles.

^b^Alternate forward primer for detection of some *L*. *noguchii* strains—especially those found in Iquitos, Peru.

^c^Alternate reverse primer for *sv*Manilae—unique among known L. interrogans/*L*. *kirschneri*/*L*. *noguchii* serotypes. *f*_GCA*/*r**_*AACT*** detect *L*. *santarosai*; *f*_GCG*/*r***_AACT*** and *f*_GTG*/*r***_ATTC*** (for some serotypes) detect *L. weilii* and *f_*GTG*/*r_***AATT*** detect L. borgpetersenii and *L. mayottensis* (some of the latter require *f*_GTG*/*r***_AACT***). Nucleotides providing amplification specificity are indicated in bold, with alleles named via reference to these polymorphic sites. For example, **AG11 = **11^th^ sequence variant amplified by AGA forward and **GTG4 = **4^th^ amplified by GTG characteristic of L. interrogans *sv*Hardjo and L. borgpetersenii *sv*Hardjo, respectively. 11108-derived amplicons are of uniform length (266 bp), with species-dependent variations in GC%, reaching a maximum of 49.2% among *L*. *santarosai*-derived amplicons and a minimum of 38.3% shared by various alleles in L. interrogans, its sister species *L*. *kirschneri*, and genetically related *L*. *noguchii*. Primers *f_***A***[*A*/*G*]***A*** (= *f***_AAA*** and *f***_AGA***) and *r_[*A*/*G*]***ACC*** (aka *r_***RA*** and *r_***RG***, respectively) detect L. interrogans and its sister species, whereas *f_***G***[*C*/*T*][*A*/*G*] and *r_***A***[*A*/*T*][*C*/*T*][*C*/*T*] amplify all other disease causing group I species. None amplify group II pathogens nor nonpathogenic species. These primer combinations are not exhaustive but as far as we know, alternate forward and reverse primers represent all sequence variants found at those locations, such screening reactions employing premixed forward and reverse primer should amplify all group-I strains **w/**expected T_m_’s.

### Detection limit, cross-species amplification of deconvoluted (i.e., species-defined) *11108* primer sets.

Genomic DNA (*g*DNA) for qPCR (and sequencing) was prepared from *interrogans sv*Lai, *santarosai sv*Shermani, and *borgpetersenii sv*Tarassovi using the manufacturer-recommended extraction protocol for Gram-negative bacteria (GeneJET DNA purification kit, Thermo Fisher Scientific) and then amplified on a CFX96 thermal cycler (Bio-Rad). Amplicons were cloned into pCR-2.1 TOPO to create species-specific reference plasmids, which were transformed into E. coli following manufacturer’s directions (TA Cloning Kit, Invitrogen). After routine minipreps (GeneJET Plasmid DNA Miniprep Kit, Thermo Fisher Scientific), purified (reference) plasmids were quantified (individually) by fluorometry (Qubit, Thermo Fisher Scientific), and their copy number estimated. This was followed by serial dilution in either PBS buffer or heat-inactivated normal human serum (NHS); re-extraction was carried out using the QIAamp DNA Blood minikit (Qiagen) per manufacturer-recommended protocol for serum, and used as template for qPCR.

As 11108 genes occur once per *Leptospira* genome, copy numbers could be expressed as genome equivalents (GEs). The detection limit was defined as minimum number of GEs producing the expected T_m_’s among all technical replicates. Reference plasmids were also used as template in amplifications evaluating the propensity of deconvoluted primer pairs for cross-species amplification.

### Serovar resolution via traditional (i.e., block-based) thermal cycling.

Reaction mixes consisted of the following: 10 μL of Precision Melt Supermix, 2 μL of template *g*DNA, and 4 μL of both forward and reverse primers, yielding final (empirically optimized) primer concentrations of 0.4 μM per 20 μL reaction. The finalized thermal cycling profile consisted of a 3-min hold at 95°C (denaturation), followed by 44 cycles of 95°C for 10 s, 62°C for 30 s (empirically optimized annealing temperature) and 72°C for 1 min (extension), and a final hold at 72°C for 7 min to ensure full extension of PCR product. Amplification was followed by melt-curve peak identification (via Precision Melt Analysis Software) with fluorescence measured at 0.2°C increments (default). All samples were run in triplicate, and runs were repeated at least three times to produce a minimum of nine data points/per sample. Cq values of ≤ 40 cycles were considered positive, but postcutoff amplification (i.e., Cq > 40) with appropriate melt peaks were recorded, and later verified by sequencing. Milli Q water was run in parallel, in triplicate, as a no template control (NTC). *g*DNA from *L*. *licerasiae Varillal sv*Varillal (archetype of group II, comprising low virulence *Leptospira* species), the genetically related *L*. *wolffii sv*Khorat, and premixed *g*DNA from Gram-negative and Gram-positive bacteria, Citrobacter rodentium (ATCC DBS 100), Escherichia coli (EPEC E2348/69), Clostridium difficile (ATCC BAA-9689), and Staphylococcus aureus (MARSA, USA 300), were used as negative controls (to assess nonspecific “off-target” amplification). For each serovar, a mean T_m_ was calculated after first identifying and then removing outlier data points via robust regression and outlier removal (ROUT) (33) analysis (Q = 10%, Prism v7.0a). Sequence-variant specific T_m_’s were defined via comparison of sample-means by two-tailed, nonparametric *t* test (*P* < 0.05, Prism v7.0a).

qPCR results were verified by conventional Sanger sequencing of cloned amplification products. Twenty-five randomly chosen colonies were subcultured into LB broth, and plasmids were isolated using a QIAprep Spin Miniprep Kit (Qiagen) and then sent for sequencing. Consensus sequences were aligned using the MAFFT multiple sequence alignment (MSA) algorithm with default parameter settings. Individual consensus sequences were submitted to the Basic Local Alignment Search Tool (BLAST) server to confirm specificity (34). For serovars consistently yielding inexact or a quasi-stable T_m_’s, we sequenced 10 randomly selected clones producing six reads per clone (for a total of 60), and then compared clone-specific consensus sequences to identify variants.

### Serovar resolution via high-precision magnetic induction thermal cycling.

Reaction mixes and thermal cycling profile were as described above, however, postamplification melt-curve analysis absorbance readings were taken at increments of 0.025°C, exploiting the superior precision of MIC. Negative controls and NTCs were run in triplicate, in parallel, as described above. And as before, outliers were removed by ROUT analysis before comparison of serovar-associated T_m_ by two-tailed, nonparametric *t* test. Data were visualized via a box-and-whisker plot.

### Reliability, accuracy, sensitivity, and specificity of *11108*-based assay.

To assess assay reliability, amplifications were done using different hardware/software combinations: (i) CFX96 with melt-curve analysis via Precision Melt Software; (ii) CFX96 with default melt-curve analysis (i.e., without Precision Melt Analysis Software); and (iii) MIC with bundled melt-curve analysis software.

To test accuracy (and thus, its potential epidemiological utility), we analyzed 45 *Leptospira* isolates obtained over a 5-year period from febrile patients, domesticated animals, and rats in Iquitos, Peru (Table S2), and 25 isolates from hospitalized patients in Sri Lanka, assessing agreement among T_m_’s, PFGE- and CAAT-derived serovar assignments. Outlier removal, statistical comparisons, and data visualization were as described above. Discrepancies among T_m_’s, PFGE and CAAT-based serotyping were resolved by Sanger-sequencing (of amplified products).

Assay sensitivity and specificity were determined using 84 canine urine samples, which comprised 40 culture positives and 44 randomly selected culture negatives and were sourced from a study of canine leptospirosis in Sokoto, Nigeria (35), as well as 12 culture-positive acute-phase serum-samples from a prospective study of human leptospirosis in Sri Lanka (36). Because *Leptospira* species and serovars in Sokoto and study sites in Sri Lanka are not well-characterized, all samples were amplified using premixed, species-appropriate primers. Shotgun metagenomics sequencing (SMS) data were generated from qPCR-positive, culture-negative samples to identify potential false-positives.

For comparison, dog urine samples were also assayed for the presence of leptospiral DNA by *lipL32* qPCR using the following forward and reverse primers: *lipL32_178f* (5′–TCT GTG ATC AAC TAT TAC GGA TAC–3′) and *lipL32_419r* (5′–ATC CAA GTA TCA AAC CAA TGT GG–3′), specific for virulent group I pathogenic species. The (previously) empirically optimized *lipL32*-based thermal cycling profile consisted of the following: 3 min denaturation at 95°C, 45 amplification cycles (95°C for 10 s/62°C for 30 s/72°C for 60 s), 7 min extension at 72°C followed by postamplification melt-peak identification. For qPCR, *g*DNA from *sv*Manilae was used as a positive control and as before, premixed *g*DNA from four bacterial species was used as a negative control.

## References

[1] Bharti AR, Nally JE, Ricaldi JN, Matthias MA, Diaz MM, Lovett MA, Levett PN, Gilman RH, Willig MR, Gotuzzo E, Vinetz JM, Peru-United States Leptospirosis Consortium. 2003. Leptospirosis: a zoonotic disease of global importance. Lancet Infect Dis 3:757–771. doi:10.1016/s1473-3099(03)00830-2. 14652202

[2] Levett PN. 2001. Leptospirosis. Clin Microbiol Rev 14:296–326. doi:10.1128/CMR.14.2.296-326.2001. 11292640 PMC88975

[3] Picardeau M. 2017. Virulence of the zoonotic agent of leptospirosis: still terra incognita? Nat Rev Microbiol 15:297–307. doi:10.1038/nrmicro.2017.5. 28260786

[4] Ko AI, Goarant C, Picardeau M. 2009. *Leptospira*: the dawn of the molecular genetics era for an emerging zoonotic pathogen. Nat Rev Microbiol 7:736–747. doi:10.1038/nrmicro2208. 19756012 PMC3384523

[5] Costa F, Hagan JE, Calcagno J, Kane M, Torgerson P, Martinez-Silveira MS, Stein C, Abela-Ridder B, Ko AI. 2015. Global morbidity and mortality of leptospirosis: a systematic review. PLoS Negl Trop Dis 9:e0003898. doi:10.1371/journal.pntd.0003898. 26379143 PMC4574773

[6] Torgerson PR, Hagan JE, Costa F, Calcagno J, Kane M, Martinez-Silveira MS, Goris MG, Stein C, Ko AI, Abela-Ridder B. 2015. Global burden of leptospirosis: estimated in terms of disability adjusted life years. PLoS Negl Trop Dis 9:e0004122. doi:10.1371/journal.pntd.0004122. 26431366 PMC4591975

[7] Hagan JE, Moraga P, Costa F, Capian N, Ribeiro GS, Wunder EA, Jr, Felzemburgh RD, Reis RB, Nery N, Santana FS, Fraga D, Dos Santos BL, Santos AC, Queiroz A, Tassinari W, Carvalho MS, Reis MG, Diggle PJ, Ko AI. 2016. Spatiotemporal determinants of urban leptospirosis transmission: four-year prospective cohort study of slum residents in Brazil. PLoS Negl Trop Dis 10:e0004275. doi:10.1371/journal.pntd.0004275. 26771379 PMC4714915

[8] Sharp TM, Rivera GB, Perez-Padilla J, Galloway RL, Guerra M, Ryff KR, Haberling D, Ramakrishnan S, Shadomy S, Blau D, Tomashek KM, Bower WA. 2016. Early indicators of fatal leptospirosis during the 2010 epidemic in Puerto Rico. PLoS Negl Trop Dis 10:e0004482. doi:10.1371/journal.pntd.0004482. 26914210 PMC4767218

[9] Yang B, de Vries SG, Ahmed A, Visser BJ, Nagel IM, Spijker R, Grobusch MP, Hartskeerl RA, Goris MG, Leeflang MM. 2019. Nucleic acid and antigen detection tests for leptospirosis. Cochrane Database Syst Rev 8:CD011871. doi:10.1002/14651858.CD011871.pub2. 31425612 PMC6699653

[10] Slack AT, Kalambaheti T, Symonds ML, Dohnt MF, Galloway RL, Steigerwalt AG, Chaicumpa W, Bunyaraksyotin G, Craig S, Harrower BJ, Smythe LD. 2008. *Leptospira wolffii* sp. *nov.*, isolated from a human with suspected leptospirosis in Thailand. Int J Syst Evol Microbiol 58:2305–2308. doi:10.1099/ijs.0.64947-0. 18842846

[11] Galloway RL, Hoffmaster AR. 2015. Optimization of LipL32 PCR assay for increased sensitivity in diagnosing leptospirosis. Diagn Microbiol Infect Dis 82:199–200. doi:10.1016/j.diagmicrobio.2015.03.024. 25912810 PMC6452440

[12] Levett PN. 2007. Sequence-based typing of leptospira: epidemiology in the genomic era. PLoS Negl Trop Dis 1:e120. doi:10.1371/journal.pntd.0000120. 18060079 PMC2100375

[13] Waggoner JJ, Pinsky BA. 2016. Molecular diagnostics for human leptospirosis. Curr Opin Infect Dis 29:440–445. doi:10.1097/QCO.0000000000000295. 27537829 PMC5127924

[14] Waggoner JJ, Balassiano I, Mohamed-Hadley A, Vital-Brazil JM, Sahoo MK, Pinsky BA. 2015. Reverse-transcriptase PCR detection of *Leptospira*: absence of agreement with single-specimen microscopic agglutination testing. PLoS One 10:e0132988. doi:10.1371/journal.pone.0132988. 26177295 PMC4503744

[15] Stoddard RA, Gee JE, Wilkins PP, McCaustland K, Hoffmaster AR. 2009. Detection of pathogenic *Leptospira* spp. through TaqMan polymerase chain reaction targeting the LipL32 gene. Diagn Microbiol Infect Dis 64:247–255. doi:10.1016/j.diagmicrobio.2009.03.014. 19395218

[16] Podgorsek D, Ruzic-Sabljic E, Logar M, Pavlovic A, Remec T, Baklan Z, Pal E, Cerar T. 2020. Evaluation of real-time PCR targeting the lipL32 gene for diagnosis of *Leptospira* infection. BMC Microbiol 20:59. doi:10.1186/s12866-020-01744-4. 32160864 PMC7066766

[17] Ahmed AA, Goris MGA, Meijer MC. 2020. Development of *lipL32* real-time PCR combined with an internal and extraction control for pathogenic *Leptospira* detection. PLoS One 15:e0241584. doi:10.1371/journal.pone.0241584. 33137154 PMC7605690

[18] Gokmen TG, Soyal A, Kalayci Y, Onlen C, Koksal F. 2016. Comparison of 16S rRNA-PCR-RFLP, *LipL32*-PCR and *OmpL1*-PCR methods in the diagnosis of leptospirosis. Rev Inst Med Trop Sao Paulo 58:64. 27680169 10.1590/S1678-9946201658064 PMC5048635

[19] Sharma KK, Kalawat U. 2008. Early diagnosis of leptospirosis by conventional methods: one-year prospective study. Indian J Pathol Microbiol 51:209–211. doi:10.4103/0377-4929.41687. 18603683

[20] Weiss S, Menezes A, Woods K, Chanthongthip A, Dittrich S, Opoku-Boateng A, Kimuli M, Chalker V. 2016. An extended multilocus sequence typing (MLST) scheme for rapid direct typing of *leptospira* from clinical samples. PLoS Negl Trop Dis 10:e0004996. doi:10.1371/journal.pntd.0004996. 27654037 PMC5031427

[21] Caimi K, Repetto SA, Varni V, Ruybal P. 2017. *Leptospira* species molecular epidemiology in the genomic era. Infect Genet Evol 54:478–485. doi:10.1016/j.meegid.2017.08.013. 28818623

[22] Rezasoltani S, Dabiri H, Khaki P, Rostami Nejad M, Karimnasab N, Modirrousta S. 2015. Characterization of *Leptospira interrogans* Serovars by polymorphism variable number tandem repeat analysis. Jundishapur J Microbiol 8:e22819. doi:10.5812/jjm.22819. 26568805 PMC4641467

[23] Guglielmini J, Bourhy P, Schiettekatte O, Zinini F, Brisse S, Picardeau M. 2019. Genus-wide *Leptospira* core genome multilocus sequence typing for strain taxonomy and global surveillance. PLoS Negl Trop Dis 13:e0007374. doi:10.1371/journal.pntd.0007374. 31026256 PMC6513109

[24] Naze F, Desvars A, Picardeau M, Bourhy P, Michault A. 2015. Use of a new high resolution melting method for genotyping Pathogenic *Leptospira* spp. PLoS One 10:e0127430. doi:10.1371/journal.pone.0127430. 26154161 PMC4496072

[25] Gregoire F, Bakinahe R, Petitjean T, Boarbi S, Delooz L, Fretin D, Saulmont M, Mori M. 2020. Laboratory diagnosis of bovine abortions caused by non-maintenance pathogenic *Leptospira* spp.: necropsy, serology and molecular study out of a Belgian experience. Pathogens 9:413. doi:10.3390/pathogens9060413. 32466444 PMC7350382

[26] Ganoza CA, Matthias MA, Saito M, Cespedes M, Gotuzzo E, Vinetz JM. 2010. Asymptomatic renal colonization of humans in the Peruvian Amazon by *Leptospira*. PLoS Negl Trop Dis 4:e612. doi:10.1371/journal.pntd.0000612. 20186328 PMC2826405

[27] Slack AT, Galloway RL, Symonds ML, Dohnt MF, Smythe LD. 2009. Reclassification of *Leptospira meyeri* serovar Perameles to *Leptospira interrogans* serovar Perameles through serological and molecular analysis: evidence of a need for changes to current procedures in *Leptospira* taxonomy. Int J Syst Evol Microbiol 59:1199–1203. doi:10.1099/ijs.0.000992-0. 19406819

[28] Levett PN. 2003. Usefulness of serologic analysis as a predictor of the infecting serovar in patients with severe leptospirosis. Clin Infect Dis 36:447–452. doi:10.1086/346208. 12567302

[29] Chirathaworn C, Janwitthayanan W, Sereemaspun A, Lertpocasombat K, Rungpanich U, Ekpo P, Suwancharoen D. 2014. Development of an immunochromatographic test with anti-LipL32-coupled gold nanoparticles for *Leptospira* detection. New Microbiol 37:201–207. 24858647

[30] Goarant C. 2014. Leptospirosis: time to move to molecular epidemiology: comments on “Reassessment of MLST schemes for *Leptospira* spp. typing worldwide” by Varni and colleagues. Infect Genet Evol 21:484–485. doi:10.1016/j.meegid.2013.10.018. 24184703

[31] Fouts DE, Brinkac L, Beck E, Inman J, Sutton G. 2012. PanOCT: automated clustering of orthologs using conserved gene neighborhood for pan-genomic analysis of bacterial strains and closely related species. Nucleic Acids Res 40:e172. doi:10.1093/nar/gks757. 22904089 PMC3526259

[32] Islam ST, Huszczynski SM, Nugent T, Gold AC, Lam JS. 2013. Conserved-residue mutations in Wzy affect O-antigen polymerization and Wzz-mediated chain-length regulation in *Pseudomonas aeruginosa* PAO1. Sci Rep 3:3441. doi:10.1038/srep03441. 24309320 PMC3854497

[33] Motulsky HJ, Brown RE. 2006. Detecting outliers when fitting data with nonlinear regression - a new method based on robust nonlinear regression and the false discovery rate. BMC Bioinformatics 7:123. doi:10.1186/1471-2105-7-123. 16526949 PMC1472692

[34] Altschul SF, Gish W, Miller W, Myers EW, Lipman DJ. 1990. Basic local alignment search tool. J Mol Biol 215:403–410. doi:10.1016/S0022-2836(05)80360-2. 2231712

[35] Pilau NN, Lubar AA, Daneji AI, Mera UM, Magaji AA, Abiayi EA, Chaiboonma KL, Busayo EI, Vinetz JM, Matthias MA. 2022. Serological and molecular epidemiology of leptospirosis and the role of dogs as sentinel for human infection in Nigeria. Heliyon 8:e09484. doi:10.1016/j.heliyon.2022.e09484. 35647333 PMC9136256

[36] Jayasundara D, Senavirathna I, Warnasekara J, Gamage C, Siribaddana S, Kularatne SAM, Matthias M, Mariet JF, Picardeau M, Agampodi S, J MV. 2021. 12 Novel clonal groups of *Leptospira* infecting humans in multiple contrasting epidemiological contexts in Sri Lanka. PLoS Negl Trop Dis 15:e0009272. doi:10.1371/journal.pntd.0009272. 33735202 PMC8009393

[37] Kokovin IL, Chernukha IG. 1970. Serological classification of leptospirae of the grippotyphosa serogroup–new serological type ratnapura. Zh Mikrobiol Epidemiol Immunobiol 47:102–105. 4251597

[38] Naotunna C, Agampodi SB, Agampodi TC. 2016. Etiological agents causing leptospirosis in Sri Lanka: a review. Asian Pac J Trop Med 9:390–394. doi:10.1016/j.apjtm.2016.03.009. 27086159

[39] Matthias MA, Ricaldi JN, Cespedes M, Diaz MM, Galloway RL, Saito M, Steigerwalt AG, Patra KP, Ore CV, Gotuzzo E, Gilman RH, Levett PN, Vinetz JM. 2008. Human leptospirosis caused by a new, antigenically unique *Leptospira* associated with a *Rattus* species reservoir in the Peruvian Amazon. PLoS Negl Trop Dis 2:e213. doi:10.1371/journal.pntd.0000213. 18382606 PMC2271056

[40] Brenner DJ, Kaufmann AF, Sulzer KR, Steigerwalt AG, Rogers FC, Weyant RS. 1999. Further determination of DNA relatedness between serogroups and serovars in the family Leptospiraceae with a proposal for *Leptospira alexanderi* sp. *nov.* and four new *Leptospira* genomospecies. Int J Syst Bacteriol 49 Pt 2:839–858. doi:10.1099/00207713-49-2-839. 10319510

