# Supplementary material for: Culture-Independent Detection and Identification of Leptospira Serovars
Source: Microbiol Spectr. 2022 Nov 29;10(6):e02475-22. doi: 10.1128/spectrum.02475-22 (PMC9769591; doi:10.1128/spectrum.02475-22)
Supplement: Supplemental file 1 — Fig. S1 and S2 and Tables S1 to S3. Download spectrum.02475-22-s0001.pdf, PDF file, 3.0 MB [file spectrum.02475-22-s0001.pdf]

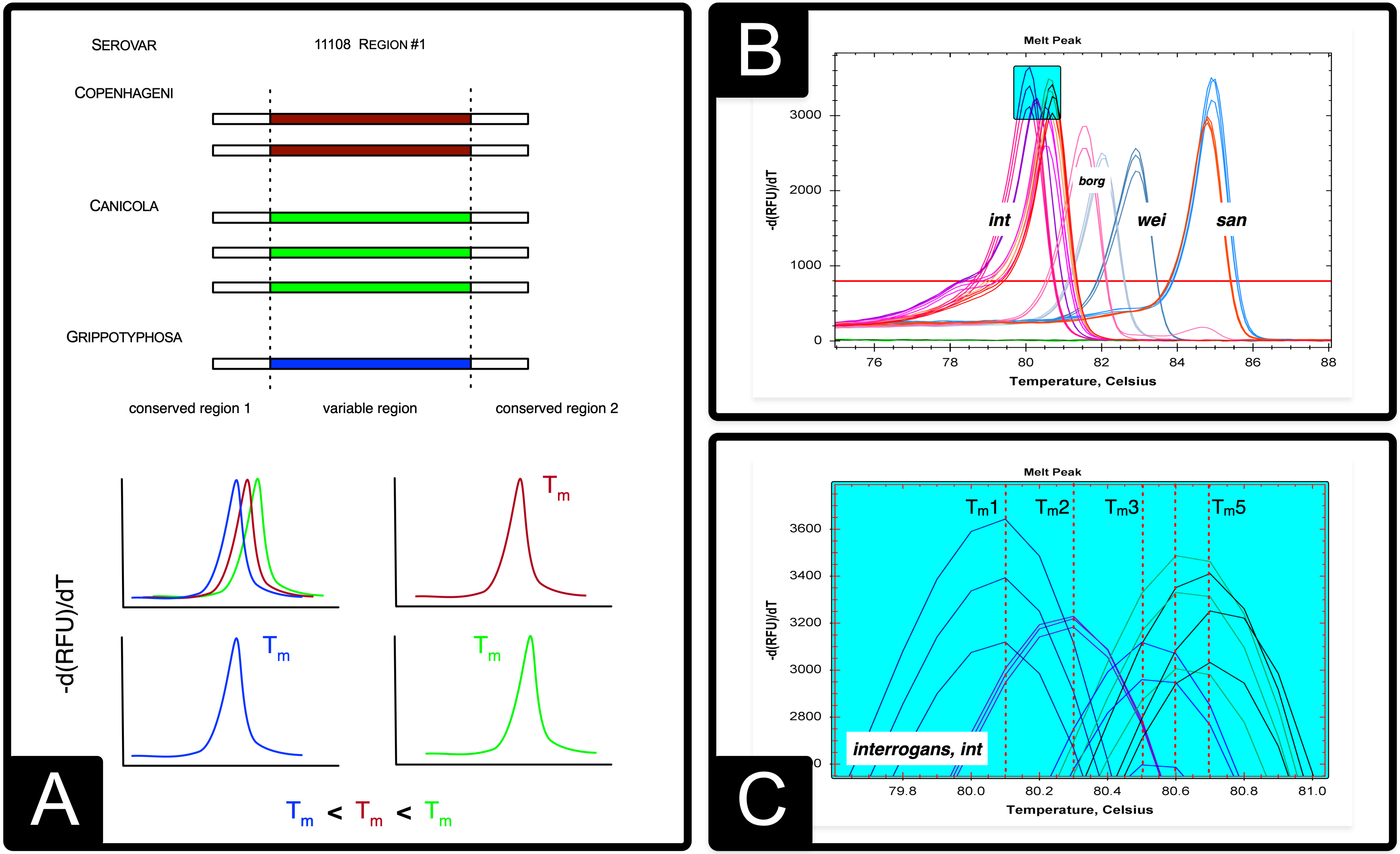

Figure S1: Assay Design and Validation. [A] Schematic illustrating 11108 assay design and anticipated outcomes. TOP: Forward and reverse primers were designed to target conserved regions (white) flanking variable regions with each color representing a hypothetical sequence variant. BOTTOM: Anticipated melt-curves from sequence variants depicted. [B] CFX96-generated melt-curves showing that melt peaks derived from individual strains belonging to the same species cluster together. *L. interrogans*, (*int*, 5 peaks enclosed in box and magnified in panel C), *L. borgpetersenii* (*borg*, 2), *L. weilii* (*wei*, 1) and *L. santarosai* (*san*, 2). Amplifications were done using degenerate primer pairs in triplicate and were repeated at least three times to assess reproducibility. [C] Melt peaks produced by five *L. interrogans* reference serovars. Serovar Icterohaemorrhagiae, *sv*Lai, *sv*Mankarso, *sv*Bratislava and *sv*Grippotyphosa (left to right).

Table S1. *Leptospira* Reference Strains

**Strain Information:** Species **Serogroup** *sv* Serovar *Strain*	**Source**
L. interrogans **Grippotyphosa** *sv* Grippotyphosa	*
L. interrogans **Icterohaemorrhagiae** *sv* Copenhageni *Fiocruz L1-130*	**
L. interrogans **Icterohaemorrhagiae** *sv* Icterohaemorrhagiae *RGA*	*
L. interrogans **Icterohaemorrhagiae** *sv* Lai *56601*	*
L. interrogans **Bataviae** *sv* Bataviae *Van Tienen*	*
L. interrogans **Autumnalis** *sv* Autumnalis *Akiyami A*	*
L. interrogans **Australis** *sv* Bratislava *Jez-Bratislava*	*
L. interrogans **Australis** *sv* Australis *Ballico*	*
L. interrogans **Sejroe** *sv* Wolffi *3705*	*
L. interrogans **Pomona** *sv* Pomona	*
L. interrogans **Pyrogenes** *sv* Pyrogenes *Salinem*	*
L. interrogans **Djasiman** *sv* Djasiman	*
L. interrogans **Mankarso** *sv* Mankarso	*
L. interrogans **Canicola** *sv* Canicola *Ruebush*	*
L. interrogans **Sejroe** *sv* Hardjo Hardjoprajitno	***
L. kirschneri **Cynopteri** *sv* Cynopteri *3522*	*
L. borgpetersenii **Ballum** *sv* Ballum *MUS 127*	*
L. borgpetersenii **Javanica** *sv* Javanica *Veldrat Bataviae 46*	*
L. borgpetersenii **Sejroe** *sv* Hardjo Hardjobovis	***
L. borgpetersenii **Tarrasovi** *sv* Tarrasovi *Perepelisin*	*
L. weilii **Celledoni** *sv* Celledoni	*
L. santarosai **Shermani** *sv* Shermani *LT 821*	**ATCC 43286**
L. santarosai **Pyrogenes** *sv* Alexi *HS 616*	*
L. santarosai **Mini** *sv* Georgia *LT 117*	*
L. santarosai **Hebdomadis** *sv* Borincana *HS 622*	*
L. licerasiae **Varillal** *sv* Varillal *VAR10*	**NR-19925**
L. wolffii **Undetermined** *sv* Korat *Korat H2T*	**NR-22250**

*Strain acquired from Renee Galloway, Centers for Disease Control and Prevention

**Strain provided as a kind gift from Dr. David Haake, University of California, Los Angeles

***Strain acquired from the USDA

Table S2. ***Leptospira*** **strains isolated over a five-year period (2002 – 2007) from humans, domesticated and wild animals, and peri-domiciliary rats in Iquitos, Peru.**

Strain	Location	Isolated	Source
CEH029	Belen	Dec-02	Rat
CEH038	Belen	Dec-02	Rat
CEH040	Belen	Dec-02	Rat
CEH242	Punchana	Feb-03	Rat
CEH243	Punchana	Feb-03	Rat
CEH246	Punchana	Feb-03	Rat
VAR033	Varillal	Mar-03	Human
BEL033	Belen	Jun-03	Human
HAI024	Hospital	May-03	Human
HAI056	Hospital	Jun-03	Human
MOR069	Moralillo	Jul-03	Human
BEL039	Belen	Aug-03	Human
BEL050	Belen	Oct-03	Human
HAI156	Hospital	Oct-03	Human
HAI257	Hospital	Nov-03	Human
VAR132U	Varillal	Nov-03	Human
PAD451	Padre.Cocha	Feb-04	Human
CBC247	Abattoir	May-04	Pig
CBC462	Abattoir	May-04	Cow
CBC297	Abattoir	Jun-04	Pig
CBC523	Abattoir	Jun-04	Cow

Strain	Location	Isolated	Source
MOR165	Moralillo	May-04	Human
MOR176BU	Moralillo	Jun-04	Human
CBC552	Abattoir	Jun-04	Cow
MMD1493	Unknown	2003	Bat
CBC613	Abattoir	Jun-04	Buffalo
CBC1203R	Abattoir	Jul-04	Pig
CBC621	Abattoir	Aug-04	Buffalo
CBC1485	Abattoir	Aug-04	Cow
HAI134	Hospital	Aug-03	Human
HAI135	Hospital	Aug-03	Human
HAI308	Hospital	Jan-04	Human
HAI645	Hospital	Oct-04	Human
HAI725	Hospital	Dec-04	Human
MMD4803	Palo.Seco	Aug-05	Possum
HAI1029	Hospital	Feb-06	Human
HAI1354	Hospital	Jan-07	Human
HAI1366	Hospital	Feb-07	Human
HAI1378	Hospital	Feb-07	Human
HAI1379	Hospital	Feb-07	Human
HAI1379U	Hospital	Mar-07	Human
HAI1378U	Hospital	Apr-07	Human
HAI1536	Hospital	Jun-07	Human
ZUN142	Zungarococha	Aug-04	Human
ZUN179	Zungarococha	Dec-04	Human

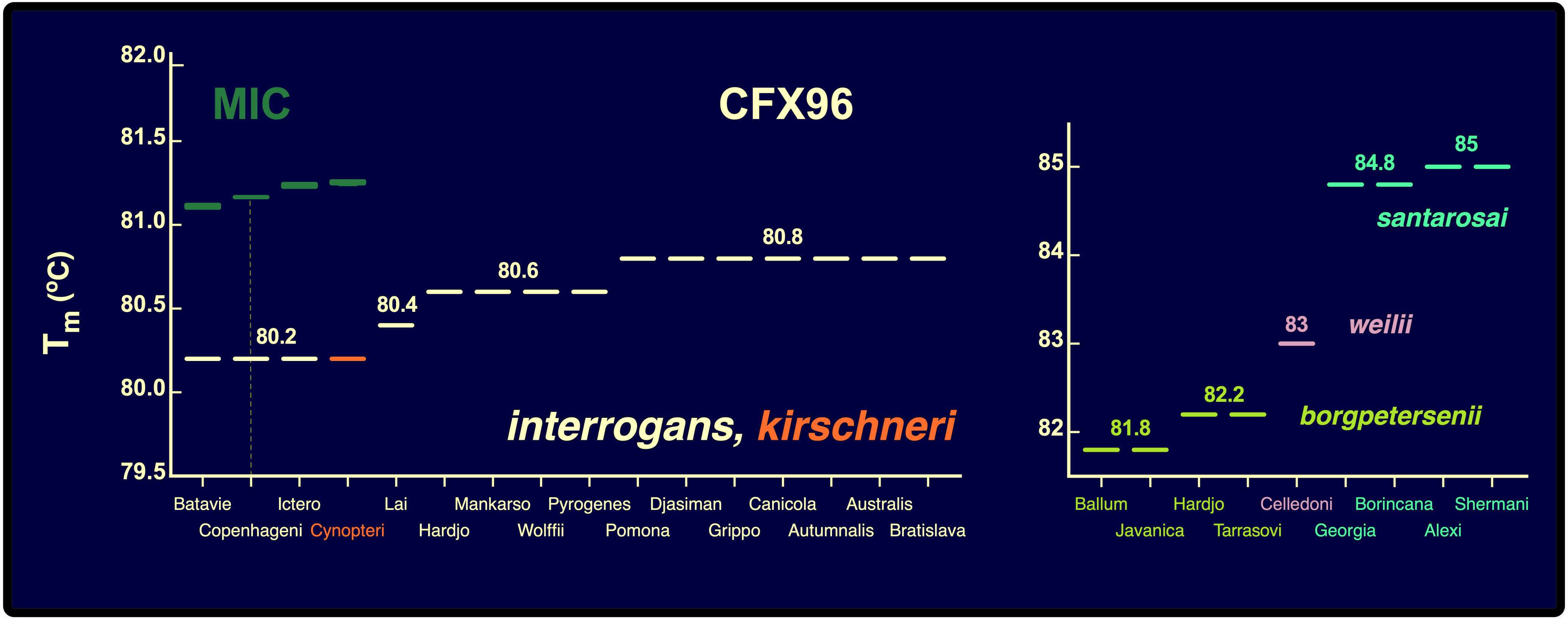

Figure S2: Box-and-whisker plots showing the mean Tm of select *Leptospira* reference strains and serovars. Data were produced from three technical replicates and three independent runs. LEFT: *L. interrogans* (15) and *L. kirschneri* (1) strains produced mean T_m's ranging from 80 – 81.5°C. Four strains (i.e., serovar Bataviae, *sv*Copenhageni, *sv*Icterohaemorrhagiae and *sv*Cynopteri) that produced a T_m of 80.2°C on the CFX96 were re-amplified by MIC platform (overlaid in green). T_m measurements were taken at increments of 0.025°C. Each serovar could be resolved from all others in the group based upon comparison of their mean T_m by two-tailed, nonparametric t-test ($\boldsymbol{p} < 0.05$, GraphPad Prism ver. 7.0a). RIGHT: *L. borgpetersenii* (4), *L. weilii* (1), and *L. santarosai* (4) strains produced a mean T_m between 81.5 – 85.5°C.

Table S3. *Leptospira* strains isolated over a fifty six years (1964-2019) period from humans in Sri Lanka. Relevant serotyping info is indicated, so too are clinical presentation.
Reference strains in bold. New alleles: **AG14**[*interrogans*], **AG15**[*kirschneri*] and **GCG2**[*weilii*]. **LIV =** liver involvement. **KID =** kidney involvement. **N/A =** Not Available.

Date	Location	Serovar/Strain	Species	Serogroup	*cg* MLST	Clonal Group	Allele	Clinical Presentation/ Outcomes**	Mean Tm	SD
**1965-1966**	**N/A**	**WEERASINGHE 6L**	*interrogans*	**Autumnalis**	**N/A**	**N/A**	AG3*	**N/A**	**N/A**	**N/A**
15/01/2018	Nelubawa	FMAS_AP1	*interrogans*	**Autumnalis**	562	266	AG3	LIV, KID	80	0.00
15/01/2018	Pahalagiribawa	FMAS_AP5	*interrogans*	**Pyrogenes**	565	10	AG10	LIV	79.8	0.00
16/02/2018	Kahatagasdigiliya	FMAS_AP6	*interrogans*	**Pyrogenes**	785	321	AG10	LIV, KID, Thrombocytopenia	80	0.00
6/3/2018	Thalawa	FMAS_AP7	*interrogans*	**Autumnalis**	786	266	AG3	LIV, KID	79.8	0.00
23/10/2017	Ilukowita	FMAS_AW1	*interrogans*	**Autumnalis**	555	74	AG3	**N/A**	79.8	0.00
22/12/2017	Kosgama	FMAS_AW2	*interrogans*	**Autumnalis**	567	269	AG3	LIV	79.8	0.00
23/12/2017	Awissawella	FMAS_AW3	*interrogans*	**Pyrogenes**	557	9	AG10	**N/A**	79.9	0.12
15/01/2018	Kegalle	FMAS_KG1	*interrogans*	**Bataviae**	560	265	AG14	LIV	80	0.00
15/01/2018	Kegalle	FMAS_KG2	*interrogans*	**Pyrogenes**	561	263	AG3	**N/A**	79.8	0.00
19/10/2017	Kithulgala	FMAS_KW1	*interrogans*	**Djasiman**	784	10	AG3	Hypotension, Thrombocytopenia, LIV, KID	79.9	0.12
23/10/2017	Bulathkohupitiya	FMAS_KW2	*interrogans*	**Autumnalis**	631	291	AG3	Hypotension, LIV	79.8	0.00
29/12/2017	Kadugannawa	FMAS_PD1	*interrogans*	**Pyrogenes**	558	263	AG3	LIV	79.7	0.12
17/08/2018	Rotawewa	FMAS_PN2	*interrogans*	**Icterohaemmorhagiae**	788	322	AG14	LIV	80	0.00
19/11/2018	Maho	FMAS_PN3	*interrogans*	**Autumnalis**	789	266	AG3	LIV	79.9	0.12
23/12/2017	Ihalaniriella	FMAS_RT2	*interrogans*	**Autumnalis**	569	271	AG3	**N/A**	79.8	0.00
**1965-1966**	**Ratnapura**	**RATNAPURA CDC**	*kirschneri*	**Grippotyphosa**	**N/A**	**N/A**	AG13*	**N/A**	**N/A**	**N/A**
**1965**	**N/A**	**GEYAWEERA CDC**	*interrogans*	**Sejroe**	**N/A**	**N/A**	AG3	**N/A**	**N/A**	**N/A**
14/01/2019	Aralaganwila	FMAS_PN5	*kirschneri*	**Grippotyphosa**	793	323	AG15	**N/A**	79.9	0.12
**1964**	**Colombo**	**CEYLONICA CDC**	*borgpetersenii*	**Javanica**	**N/A**	**N/A**	GTG3*	**N/A**	**N/A**	**N/A**
5/1/2018	Kawarakkulama	FMAS_AP2	*borgpetersenii*	**Louisiana**	563	267	GTG3	**LIV**	80.8	0.00
5/1/2018	Medawachchiya	FMAS_AP3	*borgpetersenii*	No agglutination	575	267	GTG3	**N/A**	80.6	0.00
16/02/2018	Meegalawa	FMAS_AP4	*borgpetersenii*	No agglutination	564	267	GTG3	**N/A**	80.6	0.00
14/01/2019	Galkadawala	FMAS_AP8	*borgpetersenii*	No agglutination	791	267	GTG3	**N/A**	80.7	0.12
14/01/2019	Mihinthale	FMAS_AP9	*borgpetersenii*	No agglutination	792	267	GTG3	**N/A**	80.6	0.00
7/6/2018	Wijebahupura	FMAS_PN1	*borgpetersenii*	No agglutination	787	267	GTG3	LIV	80.6	0.00
21/12/2018	Polonnaruwa	FMAS_PN4	*borgpetersenii*	No agglutination	790	267	GTG3	**N/A**	80.8	0.00
18/11/2017	Marapana	FMAS_RT1	*weilii*	No agglutination	556	262	GCG2	**N/A**	81.8	0.00
29/12/2017	Doluwa	FMAS_PD2	*weilii*	**Celledoni**	559	264	GTG6	LIV	82	0.00
**1966**	**Ratnapura**	**ALICE CDC**	*santarosai*	**Autumnalis**	**N/A**	**N/A**	GCA1*	**N/A**	**N/A**	**N/A**
